# Cell surface processing of the P1 adhesin of *Mycoplasma pneumoniae* identifies novel domains that bind host molecules

**DOI:** 10.1038/s41598-020-63136-y

**Published:** 2020-04-14

**Authors:** Michael Widjaja, Iain James Berry, Veronica Maria Jarocki, Matthew Paul Padula, Roger Dumke, Steven Philip Djordjevic

**Affiliations:** 1 The ithree institute, University of Technology Sydney, PO Box 123, Broadway, NSW 2007 Australia; 2 Technische Universität Dresden, Medizinische Fakultät Carl Gustav Carus, Institut für Medizinische Mikrobiologie und Hygiene, Fetscherstrasse 74, 01307 Dresden, Germany; 3 Proteomics Core Facility and School of Life Sciences, University of Technology Sydney, PO Box 123, Broadway, NSW 2007 Australia

**Keywords:** Proteomic analysis, Proteolysis, Bacterial pathogenesis, Pathogens

## Abstract

*Mycoplasma pneumoniae* is a genome reduced pathogen and causative agent of community acquired pneumonia. The major cellular adhesin, P1, localises to the tip of the attachment organelle forming a complex with P40 and P90, two cleavage fragments derived by processing Mpn142, and other molecules with adhesive and mobility functions. LC-MS/MS analysis of *M*. *pneumoniae* M129 proteins derived from whole cell lysates and eluents from affinity matrices coupled with chemically diverse host molecules identified 22 proteoforms of P1. Terminomics was used to characterise 17 cleavage events many of which were independently verified by the identification of semi-tryptic peptides in our proteome studies and by immunoblotting. One cleavage event released ^1597^TSAAKPGAPRPPVPPKPGAPKPPVQPPKKPA^1627^ from the C-terminus of P1 and this peptide was shown to bind to a range of host molecules. A smaller synthetic peptide comprising the C-terminal 15 amino acids, ^1613^PGAPKPPVQPPKKPA^1627^, selectively bound cytoskeletal intermediate filament proteins cytokeratin 7, cytokeratin 8, cytokeratin 18, and vimentin from a native A549 cell lysate. Collectively, our data suggests that ectodomain shedding occurs on the surface of *M*. *pneumoniae* where it may alter the functional diversity of P1, Mpn142 and other surface proteins such as elongation factor Tu via a mechanism similar to that described in *Mycoplasma hyopneumoniae*.

## Introduction

The attachment organelle is a structurally and functionally sophisticated component of the *M*. *pneumoniae* cell that is responsible for the assembly of proteins essential for motility and adherence^1–8^. An extensive list of host molecules including fibronectin^9–13^, fibrinogen^10–14^, plasminogen^11–13,15–17^, lactoferrin^10–12^, laminin^10–12^, and vitronectin^10–13^ interact with surface accessible adhesins in *M*. *pneumoniae*. Other less well-defined host molecules include sialylated molecules^18^, oligosaccharides^19^, glycolipids^20^, and glycoproteins^21^.

The gene *mpn141* encoding the major adhesin P1 is located in the same operon along with *mpn140* and *mpn142* and these three genes constitute a polycistronic transcriptional unit^22,23^. *mpn140* encodes for a 28 kDa putative phosphoesterase^24^ and while it has been shown to degrade nanoRNA and dephosphorylate 3′-phosphoadenosine 5′-phosphate to AMP^25^, no role in adherence has been assigned for this protein. *mpn142* generates a 130 kDa product (Mpn142) that is cleaved into two fragments of 40 kDa (P40) and 90 kDa (P90) immediately after or concurrent with translation^26,27^. The cleavage event in Mpn142, first described over 25 years ago, was the first in what is now known to be a highly processed molecule on the surface of *M*. *pneumoniae*^28^. P1 is a remarkably versatile molecule and the subject of numerous studies over the past 30 years. The only cleavage event that has been accurately assigned to P1 is the removal of the N-terminal 59 amino acids as a leader peptide^29^. Molecular cross-linking and immunogold-labelling studies indicated that P1 forms a complex with P30, P40, and P90^30,31^ that colocalise to the tip of the attachment organelle to act in concert to effect different functions^5,6,23,32^. Cross-linking studies with paraformaldehyde identified P1 complexes containing Mpn309 (P65), Mpn272 (DnaK), C-terminal truncated forms of DnaK and P1, pyruvate dehydrogenase α subunit (Pdh-A), and high molecular weight proteins 1 (HMW1) and 3 (HMW3)^33^. Anti-P1 antibodies reduce adherence of *M*. *pneumoniae* to abiotic and host cell surfaces^3,34–38^ and *M*. *pneumoniae* P1 mutants are also unable to adhere^35,36,39–41^. For P1 to translocate to the surface, localise correctly within the attachment organelle and to maintain stability, interactions with accessory proteins P40, P90, HMW1, and TopJ are required^2,42–46^. C-terminal regions of P1 have featured in various recombinant vaccines that seek to control infections caused by *M*. *pneumoniae*.

The P1 adhesin is highly immunogenic and is often detected by sera from *M*. *pneumoniae-*infected patients^36,47,48^. Several studies have shown that the carboxyl half of P1 is highly immunogenic and crucial for its function as an adhesin^37,49–55^. To identify regions within P1 that are recognised by the host humoral immune response, Schurwanz *et al*. generated 15 recombinant fragments spanning the P1 molecule and exposed them to the serum of patients with *M*. *pneumoniae* infections^55^. Three recombinant fragments within P1, one in the N-terminus and two spanning C-terminal regions, were strongly immunoreactive with sera from greater than 90% of the patients^55^. Guinea pig antibodies generated to one of the C-terminal regions significantly reduced binding of *M*. *pneumoniae* to HBEC (primary bronchial epithelial), MRC-5 (fetal lung fibroblasts), and HeLa (cervical carcinoma) cell lines^55^. These data informed the creation of a chimeric recombinant protein which included this carboxyl region of P1 and a region in the P30 adhesin. Antibodies raised against this chimeric protein reduced *M*. *pneumoniae* adherence to human bronchial epithelial cells by more than 95%^55^, and also successfully reduced *M*. *pneumoniae* colonisation in animal models^56^.

Here we sought to determine if P1 is processed on the surface of *M*. *pneumoniae*. Tryptic peptides that mapped to different regions within P1 were frequently encountered when characterising size-fractionated eluents generated during affinity chromatography using different host molecules as bait. These peptides were mapped to the P1 molecule providing insight into the complex processing events that target this molecule. Precise cleavage sites were determined using an N-terminome approach^57^ and by mapping semi-tryptic peptides identified from our proteome studies. Naturally occurring cleavage fragments of P1 were identified by LC-MS/MS analysis i) because they bound to affinity resins loaded with host proteins, ii) by mapping tryptic peptides derived from proteins spots from 2D-SDS PAGE, and iii) by immunoblotting studies using serum raised against fifteen different regions of P1. These independently acquired, but complementary datasets enabled a rigorous assessment of cleavage events in the P1 adhesin. Finally, microtitre binding assays and microscale thermophoresis showed that the C-terminus of P1 binds various host molecules.

## Methods and Materials

### Strains

*M*. *pneumoniae* (M129 strain, ATCC 29342) cells were cultured as described previously^58^. Cells were grown in modified Hayflick’s medium in tissue culture flasks at 37 °C. Human lung carcinoma (A549, ATCC CCL-185) cells were cultured in RPMI 1640 medium (Invitrogen) supplemented with 10% heat inactivated fetal bovine serum. Cells were grown in tissue culture flasks at 37 °C with 5% CO_2_.

### Cell preparation for one dimensional- and two dimensional-SDS polyacrylamide gel electrophoresis

*M*. *pneumoniae* cells were harvested as described previously^59^. In brief, cells were lysed with sonication in 7 M urea, 2 M thiourea, 40 mM Tris-HCl, and 1% (w/v) C7BzO detergent (Sigma) after washing with PBS. Proteins were reduced and alkylated with 5 mM tributylphosphine and 20 mM acrylamide monomers before precipitation with acetone. Protein was resuspended in 7 M urea, 2 M thiourea, and 1% (w/v) C7BzO for 1D- and 2D-SDS PAGE.

Gel electrophoresis was performed as described previously^60,61^. Approximately 80 µg and 250 µg of protein was used for 1D- and 2D-SDS PAGE, respectively. Gels were fixed and stained by either Flamingo fluorescent gel stain (Bio-Rad) or Coomassie Blue G-250 (Sigma).

In-gel trypsin digestion was performed as described previously^62^ for mass spectrometry analysis. Gel pieces were excised, destained, dehydrated, and then incubated with trypsin Gold MS grade (Promega) in 100 mM NH_4_HCO_3_. Tryptic peptides were extracted by sonication and stored in 4 °C until needed for mass spectrometry.

### Liquid chromatography tandem mass spectrometry (LC-MS/MS) and data analysis

LC-MS/MS was performed as described previously^61^. In brief, 5 μg of peptides in 15 μl was loaded into an Eksigent AS-1 autosampler connected to a Tempo nanoLC system (Eksigent, Livermore, CA, USA) and washed onto a PicoFrit column (75 μm × 150 mm) packed with Magic C18AQ resin (Michrom Biosciences, CA). Peptides were eluted from the column into the source of a QSTAR Elite hybrid quadrupole-time-of-flight mass spectrometer (Sciex, Redwood, CA, USA).

Files generated from LC-MS/MS were searched against the MSPnr100 database^63^ with the following parameters: Fixed modifications: none; Variable modifications: propionamide, oxidized methionine, deamidation; Enzyme: semi-trypsin; Number of allowed missed cleavages: 3; Peptide mass tolerance: 100 ppm; MS/MS mass tolerance: 0.2 Da; and Charge state: 2+, 3+, and 4+. For samples collected from the ‘Surface proteome analysis of *M*. *pneumoniae* (Biotinylation)’ and ‘Affinity chromatography host binding *M*. *pneumoniae* complexes (A549)’ listed below, variable modifications also included NHS-LC-Biotin (K) and NHS-LC-Biotin (N-term). ‘Affinity chromatography host binding *M*. *pneumoniae* complexes (A549)’ was also searched against *‘homo sapiens’* entries in MSPnr100 to identify biotinylated surface A549 proteins.

### Surface proteome analysis of M. pneumoniae

Biotinylation of the *M*. *pneumoniae* cells was performed as described previously^28^. The biotinylation reaction was allowed to proceed for 30 seconds on ice. Biotinylated surface proteins were confirmed with western blots using ExtrAvidin-HRP (Sigma).

Trypsin shaving of *M*. *pneumoniae* cells was carried out as described previously^12^. Shaving was for 5 minutes at 37 °C and released peptides were trypsin digested a second time before analysis by LC-MS/MS.

### Affinity chromatography of host binding *M. pneumoniae* complexes

‘Bait’ host proteins used for affinity chromatography include fibronectin (Code: 341635) and plasminogen (Code: 528175) from human plasma supplied by Merck Millipore. Bovine actin (Code: A3653) and fetuin (Code: F3004) was supplied by Sigma.

Affinity chromatography using host proteins bound to Avidin Agarose (Pierce) as ‘Bait’ was performed as described previously^28^. *M*. *pneumoniae* cells were lysed in 1% (w/v) C7BzO (Sigma-Aldrich) in PBS (pH 7.8) to obtain native complexes. The native complex cell lysate was incubated with host proteins bound to Avidin Agarose (‘Bait’). This mixture was washed with PBS and host protein binding complexes (‘Prey’) were eluted 7 M urea, 2 M thiourea, 40 mM Tris-HCl, and 1% (w/v) C7BzO. Elutions were separated by 1D-SDS PAGE and proteins were identified by LC-MS/MS as described above.

Affinity chromatography using human lung carcinoma (A549) surface proteins as ‘Bait’ was performed as described previously^28^. A549 cells were biotinylated, lysed, and bound to Avidin Agarose (‘Bait’). As above, this mixture was incubated with native *M*. *pneumoniae* complexes followed by washes and eluents to obtain a fraction of A549 binding complexes (‘Prey’).

Affinity chromatography using heparin HiTrap columns (GE Healthcare) was performed as described previously^28^. *M*. *pneumoniae* cells were lysed in 10 mM sodium phosphate, 0.1% Triton TX-100 (pH 7.0) to obtain native complexes. Approximately 300 µg of soluble complexes was loaded onto a HiTrap Heparin HP column (GE Healthcare). The column was washed with 10 mM sodium phosphate (pH 7.0) and heparin binding complexes were sequentially eluted in increasing concentrations of sodium chloride (pH 7.0).

### Dimethyl labelling of M. pneumoniae and LC-MS/MS analysis

Dimethyl labelling of *M*. *pneumoniae* proteins was carried out as described previously^28,64^. 1 mg of *M*. *pneumoniae* protein was labelled in 40 mM formaldehyde (Ultrapure grade, Polysciences), 20 mM sodium cyanoborohydride, 100 mM Hepes (pH 6.7) for 4 hours at 37 °C. The reaction was quenched with 100 mM ammonium bicarbonate, precipitated in acetone:methanol (8:1), and digested with trypsin.

Peptides were analysed using both the Sciex 5600 and Thermo Scientific Q Exactive™ mass spectrometers. The methods, protocols, and parameters used have been described previously^28^.

### Bioinformatic analysis of the P1 adhesin

Bioinformatic predictions and analysis was performed as described previously^28^. The bioinformatic tools used were: ProtParam^65^, TMpred^66^, PONDR® (VSL2 predictor)^67^, and ScanProsite^68^. Predicted glycosaminoglycan binding motifs searched in ScanProsite included binding sites for heparin (X-[HRK]-[HRK]-X-[HRK]-X motif)^69^, heparin sulfate (X-[HRK]-X-[HRK]-[HRK]-X)^70^, or clusters of basic amino acid residues (X-[HRK]-X(0,2)-[HRK]-X(0,2)-[HRK]-X and X-[HRK]-X(1,3)-[HRK]-X(1,3)-[HRK]-X).

### Immunoblot of M. pneumoniae cell lysates using Anti-P1 serum

60 µg of *M*. *pneumoniae* cell lysate proteins were separated on 1D-SDS PAGE as described above. Proteins were transferred to PVDF (polyvinylidene fluoride) membranes using a semidry method^71^. Membranes were blocked with 5% (w/v) skim milk powder in PBS, and 0.1% (v/v) Tween 20 (PBS-Tween) for 1 hour at 25 °C. Membranes were cut in to individual lanes and then separately probed with guinea pig sera raised against different regions of the P1 adhesin (guinea pig sera was generated in a previous study^55^) for 1.5 hours at 25 °C in PBS-Tween. Membranes were washed three times over 30 minutes before being probed a second time in peroxidase-conjugated anti-guinea pig antibodies (1:3000, Sigma) for 1 hour at 25 °C in PBS-Tween. Membranes were washed again three times over 30 minutes and developed with DAB tablets (3,3′-Diaminobenzidine, Sigma).

### Binding of P1 C-terminus to human proteins in ELISA

Human proteins used for ELISA include: plasma fibrinogen (Code: F3879), plasma fibronectin (Code: 11051407001), Glu-plasminogen (Code: P7999), vitronectin (Code: SRP3186), laminin (Code: L6274), and lactoferrin (Code: L1294) which were all supplied by Sigma.

Binding affinity measured by ELISA was performed as described previously^17^. Recombinant protein RP15 was produced as described^55^ and both C-terminal peptides were synthesised by Chempeptide Limited (China). P1-30 (^1597^TSAAKPGAPRPPVPPKPGAPKPPVQPPKKPA^1627^) without any tags, but P1-15 (^1613^PGAPKPPVQPPKKPA^1627^) was sequenced with an N-terminal biotin tag.

15 µg/ml of C-terminal P1 fragments were bound to wells and incubated with different host proteins. Wells were then incubated with different antiserum raised against the different host proteins at the following dilutions (all from Sigma): anti-fibrinogen 1:3000, anti-fibronectin 1:1000, anti-plasminogen: 1:2500, anti-vitronectin 1:5000, anti-laminin 1:750, and anti-lactoferrin 1:5,000. These incubations were followed by incubations with anti-rabbit IgG (Dako) or anti-goat IgG (both 1:2,000). Detection was measured by adding Tetramethylbenzidine (Sigma) followed by 1 M HCl, and absorbance was measured at 450 nm (620 nm as reference).

### Binding of the P1 C-terminus to A549 human lung cells

Freshly grown A549 cells were immobilised in 96-well microtitre plates as described in^17^. Immobilised A549 cells were incubated with 10 µg/ml of either RP15, P1-30, or P1-15 and binding affinity was measured with antiserum raised against RP15 (1:100) as described above. Absorbance detection at 450 nm is the same as described above.

### Affinity chromatography of complexes that bind the P1 C-terminus

The C-terminal sequence of P1 (P1-15) was synthesised with an N-terminal biotin tag by Chempeptide Limited (China). Affinity chromatography was performed similar to the section above. In brief, 1 mg of the peptide was added to Avidin Agarose beads for 16 h at 4 °C. The beads were washed four times (5 ml per wash) with PBS before being incubated with native A549 cell lysates (harvested in 1% w/v C7BzO in PBS) for 16 h at 4 °C. Non-binding proteins were washed from the column with four washes (5 ml per wash) of PBS and protein complexes with an affinity to the peptide were eluted from the column with 7 M urea, 2 M thiourea, 40 mM Tris-HCl, and 1% (w/v) C7BzO (4 times of 2 ml). Eluents were concentrated with a Macrosep® 3 kDa cutoff centrifugal device (Pall), precipitated with acetone, and separated by 1D-SDS PAGE. The whole lane was divided into sections, in-gel digested with trypsin, and analysed by LC-MS/MS as described above.

### Microscale thermophoresis of P1-15 binding affinity

Binding affinities to fluorescent labelled host proteins was measured by microscale thermophoresis as described in^72^. Microscale thermophoresis was set to 30 s and samples were scanned with 40%, 60% and 80% MST Power. Dissociation constants were determined from generated dissociation curves with set hot/cold or thermophoresis settings. As a control, a scrambled version of the C-terminal P1 peptide (PKPPRAAPPKAPTPVPPGPASPVKKPKQAPG) was synthesised by Chempeptide Limited (China) without any tags and binding affinities was measured.

### Ethical approval

Guinea pig sera used in this study was generated in a previous study^55^. The animal experiments in that previous study were proved by the ethical board of Landesdirektion Sachsen, Dresden, Germany (no. 24-9168.25-1).

## Results

### Bioinformatic analysis of the P1 adhesin

**Figure 1:**
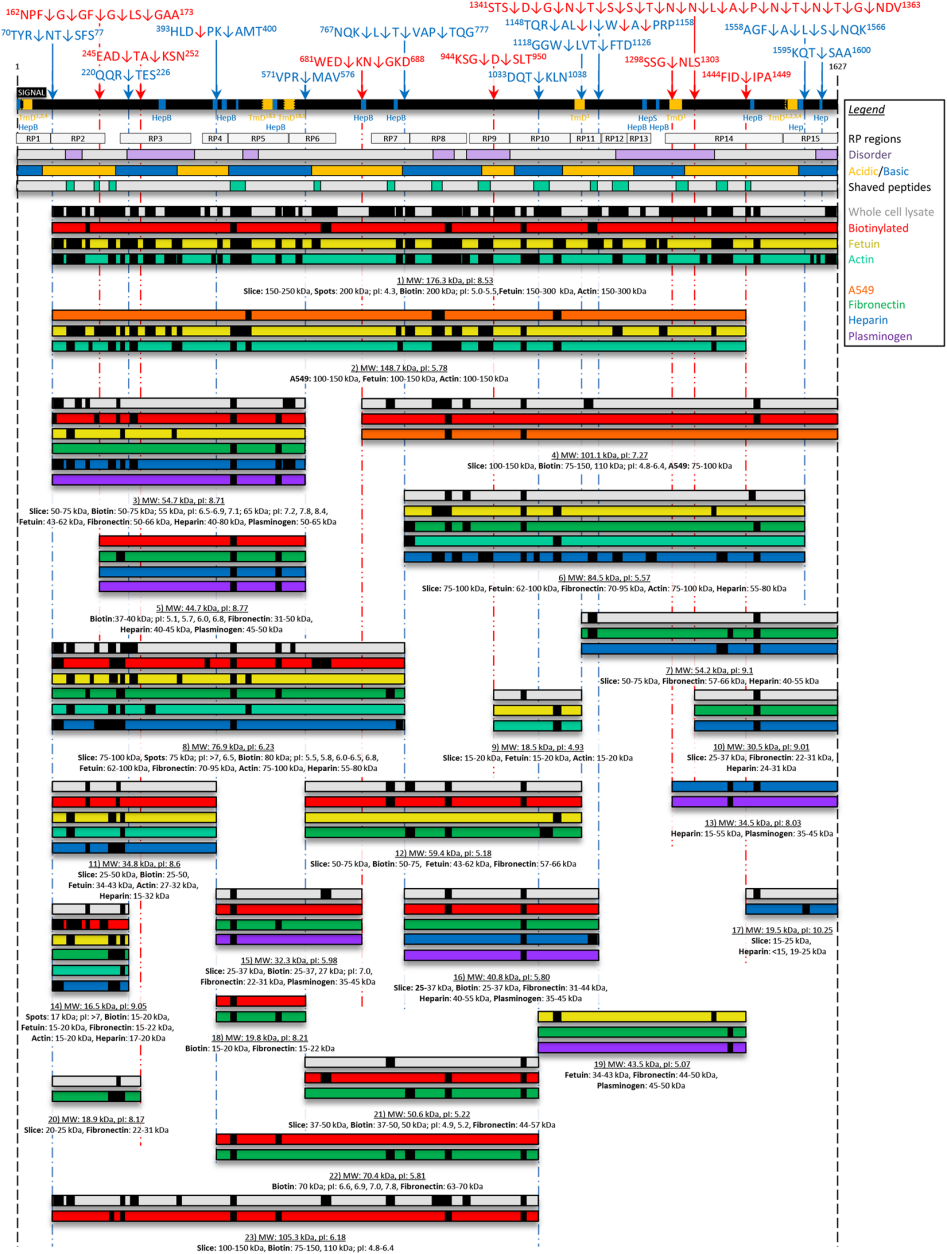
Cleavage map of the P1 adhesin. The full length proteoform (1627 amino acids) is shown as the black bar with cleavage sites above and fragments below this bar. Cleavage sites identified from dimethyl labelling and semi-tryptic sites are shown as the blue and red arrows, respectively. Sequences where these cleavage sites occur are also shown. Putative heparin binding sites (Hep, blue boxes, motif: X-[HRK]-[HRK]-X-[HRK]-X), heparan sulfate binding sites (Hep^S^, blue boxes, motif: X-[HRK]-X-[HRK]-[HRK]-X), clusters of basic residues (Hep^B^, blue boxes, motifs: X-[HRK]-X(0,2)-[HRK]-X(0,2)-[HRK]-X or X-[HRK]-X(1,3)-[HRK]-X(1,3)-[HRK]-X), and transmembrane domains (TmD, yellow boxes, TmD^1^ predicted by TMpred^66^, TmD^2^ previously predicted^32^, TmD^3^ previously predicted^36^, and TmD^4^ previously predicted in a P1 paralog^73^) are shown within the black bar. Putative transmembrane domains and the location of 15 subregions of P1 (grey ‘RP’ boxes) expressed as recombinant proteins from an earlier study^55^ are shown. Predicted disordered regions appear as purple boxes in the grey bar. Acidic and basic regions within P1 are identified as yellow and blue bars, respectively. Peptides released from surface shaving experiments and identified by mass spectrometry are shown in the light green boxes within the grey bar. Grey bars represent fragments of P1 identified during SDS-PAGE of whole cell lysates. Red bars represent fragments of P1 recovered from lysates of *M*. *pneumoniae* that have their surface proteins labelled with biotin (surface exposed fragments of P1). Peptides identified by mass spectrometry of P1 fragments isolated from affinity chromatography of fetuin (yellow bars), actin (light blue bars), A549 surface protein complexes (orange bars), fibronectin (green bars), heparin (blue bars), and plasminogen (purple bars) are shown.

The P1 adhesin has a predicted mass of 176.3 kDa and a p*I* of 8.53 and contains six predicted transmembrane regions and nine putative glycosaminoglycan binding sites (Fig. 1). The first transmembrane region (spanning the N-terminus), and the last transmembrane region (spanning the C-terminus) have been identified in previous studies of P1^32,36,66^, and a P1 paralog of *Mycoplasma genitalium*^73^. The glycosaminoglycan binding sites consist of reiterated copies of positively charged amino acids that are likely to be important in interactions with sulphated derivatives of heparin and heparan sulfate. Analysis of P1 using PONDR® identified seven putative disordered regions that span at least 30 amino acids (Fig. 1). Modules in P1 enriched in acidic (E, K) and basic (K, R, H) amino acids were identified. Disordered region and protein modules enriched in acidic and basic amino acids have been described in adhesin families in the respiratory pathogen *M*. *hyopneumoniae* and these were influential in the location of a subset of important cleavage sites^60–62,74^. We confirmed the precise location of 17 cleavage sites in P1 (shown below), 11 of which reside in predicted regions of disorder (Fig. 1). Cleavage sites did not seem to be over-represented in acidic or basic domains.

### The P1 adhesin is processed extensively on the M. pneumoniae cell surface

P1 peptides identified by LC-MS/MS analyses of size fractionated *M*. *pneumoniae* lysates identified 23 proteoforms ranging in size from 17 to 176 kDa including the full length proteoform without the N-terminal signal sequence (Fig. 1). The full length and an additional 16 smaller proteoforms of P1 were identified by LC-MS/MS of size fractionated cell lysates separated by SDS-PAGE (grey bars; Fig. 1). The migration behaviour of these 17 proteoforms of P1 was consistent with masses predicted by ProtParam^65^. Trypsin shaving of the *M*. *pneumoniae* cell surface released trypsin accessible peptides (green boxes within a grey bar in Fig. 1) that span most of the adhesin indicating that P1 is exposed on the cell surface. This was consistent with LC-MS/MS analysis of size-fractionated biotinylated proteins that were first enriched using avidin chromatography which identified 14 proteoforms (full and fragments 2, 3, 5, 7, 10, 11, 13, 14, 16, 17, 20, 21, and 22) of P1 (red bars in Fig. 1). These data suggest that cleaved P1 proteoforms are surface accessible.

Several other proteoforms of P1 were identified by LC-MS/MS of protein bands digested in-gel from affinity experiments. Two proteoforms of P1 with masses of 149 kDa (fragment 1) and 101 kDa (fragment 3) (orange bars in Fig. 1) were identified from columns coupled with biotinylated A549 surface protein complexes suggesting that large P1 proteoforms with multiple binding domains are required to bind surface receptors on A549 cells. Eluents derived from columns coupled with fetuin and actin were particularly useful for identifying the full length protein and fragments 1, 2, 4, 7, 8, 10, 11, 16 and 18 of P1. Nine fragments (1, 2, 4, 7, 8, 10, 11, 16, and 18; yellow bars in Fig. 1) were recovered from columns coupled with fetuin, and six fragments (1, 4, 7, 8, 10, and 16; light blue bars in Fig. 1) were recovered from columns coupled with actin. Six fragments (2, 5, 12, 13, 14, 18) were identified from columns coupled with plasminogen (purple bars in Fig. 1). For the eleven fragments identified from heparin chromatography (blue bars in Fig. 1; fragments: 2, 4, 5, 6, 7, 9, 10, 12, 14, 15, and 16), only two (fragment 14 and 16) did not contain any of the predicted glycosaminoglycan binding motifs identified with ScanProsite. Fifteen fragments (2, 4, 5, 6, 7, 9, 11, 13, 14, 16, 17, 18, 19, 20, and 21) were identified in eluents from columns coupled with fibronectin (green bars in Fig. 1).

A global *M*. *pneumoniae* dimethyl labelling approach was used to identify internal neo-N termini. Ten cleavage sites were identified in P1 using this approach (Table 1, blue arrows in Fig. 1). Semi-tryptic peptides, defined as peptides with only one tryptic end (Table 1, red arrows in Fig. 1) were also identified, implying seven additional cleavage sites in P1. Four distinct sites in P1 showed evidence that surface accessible amino-peptidases may alter neo-N-termini (Fig. 1 and Table 1): ^162^NPF↓G↓GF↓G↓LS↓GAA^173^ (cleavage site 2), ^767^NQK↓L↓T↓VAP↓TQG^777^ (cleavage site 8), ^1148^TQR↓AL↓I↓W↓A↓PRP^1158^ (cleavage site 12), and ^1558^AGF↓A↓L↓S↓NQK^1566^ (cleavage site 16) in a manner that is similar to amino-peptidase processing events reported in the major adhesin families in *M*. *hyopneumoniae*^57,64,74,75^. A large predicted disorder region spanning 196 amino acids near the carboxyl terminal of P1 represents a fifth site for high amino-peptidase activity with 18 neo-N-termini residing between amino acid positions 1343 – 1361 (cleavage site 14 in Table 1; sequence: ^1341^STS↓D↓G↓N↓T↓S↓S↓T↓N↓N↓L↓A↓P↓N↓T↓N↓T↓G↓NDV^1363^).

**Table 1:** N-terminal dimethylated peptides identified in P1 adhesin by LC-MS/MS.

No.	ID	Peptide Sequence	Score	E-value
		N-terminal dimethyl labelled peptides		
1	N1	R.^73^**N**TSFSSLPLTGENPGAWALVR^93^.D	107	6.20E^-09^
	N2	T.^75^**S**FSSLPLTGENPGAWALVR^93^.D	26	0.039
3	N3	R.^224^**T**ESGQNTSTTGAMFGLKVKNAEADTAKSNEKLQGAEATGSSTTSGSGQSTQR^275^.G	72	6.60E^-07^
5	N4	K.^398^**A**MTANYPPSWR^408^.T	67	5.70E^-05^
6	N5	R.^574^**M**AVAGAKFVGR^584^.E	62	5.00E^-04^
8	N6	K.^770^**L**TVAPTQGTNWSHFSPTLSR^789^.F	121	7.00E^-11^
	N7	L.^771^**T**VAPTQGTNWSHFSPTLSR^789^.F	77	8.10E^-07^
	N8	T.^772^**V**APTQGTNWSHFSPTLSR^789^.F	64	1.00E^-05^
	N9	P.^775^**T**QGTNWSHFSPTLSR^789^.F	35	5.30E^-03^
10	N10	T.^1036^**K**LNLPAYGEVNGLLNPALVETYFGNTR^1062^.A	173	7.10E^-16^
11	N11	W.^1121^**L**VTFTDFVKPR^1131^.A	58	7.20E^-05^
	N12	T.^1124^**F**TDFVKPR^1131^.A	40	5.70E^-03^
12	N13	R.^1151^**A**LIWAPRPWAAFR^1163^.G	36	1.20E^-03^
	N14	I.^1154^**W**APRPWAAFR^1163^.G	27	2.10E^-03^
16	N15	F.^1561^**A**LSNQKVDVLTKAVGSVFKEIINR^1584^.T	160	1.70E^-14^
	N16	A.^1562^**L**SNQKVDVLTKAVGSVFKEIINR^1584^.T	158	1.30E^-13^
	N17	L.^1563^**S**LSNQKVDVLTKAVGSVFKEIINR^1584^.T	184	3.90E^-16^
	N18	S.^1564^**N**QKVDVLTKAVGSVFKEIINR^1584^.T	102	4.60E^-09^
17	N19	T.^1598^**S**AAKPGAPRPPVPPKPGAPKPPVQPPKKPA^1627^	58	4.50E^-06^
		N-terminal semi-tryptic peptides		
2	S1	F.^165^**G**GFGLSGAAPQQWNEVKNKVPVEVAQDPSNPYR^197^.F	39	2.10E^-03^
	S2	G.^166^**G**FGLSGAAPQQWNEVKNKVPVEVAQDPSNPYR^197^.F	34	3.20E^-03^
	S3	F.^168^**G**LSGAAPQQWNEVKNKVPVEVAQDPSNPYR^197^.F	42	0.037
	S4	G.^169^**L**SGAAPQQWNEVKNKVPVEVAQDPSNPYR^197^.F	59	5.00E^-06^
	S5	S.^171^**G**AAPQQWNEVKNKVPVEVAQDPSNPYR^197^.F	46	3.0E^-04^
4	S6	D.^248^**T**AKSNEKLQGAEATGSSTTSGSGQSTQR^275^.G	126	1.00E^-11^
	S7	A.^250^**A**KSNEKLQGAEATGSSTTSGSGQSTQR^275^.G	80	4.20E^-07^
5	S8	D.^396^**P**KAMTANYPPSWR^408^.T	103	1.40E^-08^
7	S9	D.^684^**K**NGKDDAKYIYPYR^694^.Y	58	1.90E^-04^
	S10	N.^686^**G**KDDAKYIYPYR^694^.Y	61	2.00E^-04^
12	S11	L.^1153^**I**WAPRPWAAFR^1163^.G	56	2.00E^-04^
	S12	I.^1156^**W**APRPWAAFR^1163^.G	46	3.90E^-03^
	S13	W.^1154^**A**PRPWAAFR^1163^.G	49	3.30E^-04^
	S14	A.^1155^**P**RPWAAFR^1163^.G	46	3.10E^-03^
14	S15	S.^1344^**D**GNTSSTNNLAPNTNTGNDVVGVGR^1368^.L	90	1.20E^-08^
	S16	D.^1345^**G**NTSSTNNLAPNTNTGNDVVGVGR^1368^.L	192	8.00E^-17^
	S17	G.^1346^**N**TSSTNNLAPNTNTGNDVVGVGR^1368^.L	158	1.20E^-13^
	S18	N.^1347^**T**SSTNNLAPNTNTGNDVVGVGR^1368^.L	154	6.00E^-13^
	S19	T.^1348^**S**STNNLAPNTNTGNDVVGVGR^1368^.L	121	7.90E^-10^
	S20	S.^1349^**S**TNNLAPNTNTGNDVVGVGR^1368^.L	126	1.90E^-10^
	S21	S.^1350^**T**NNLAPNTNTGNDVVGVGR^1368^.L	121	4.60E^-11^
	S22	T.^1351^**N**NLAPNTNTGNDVVGVGR^1368^.L	117	4.20E^-10^
	S23	N.^1352^**N**LAPNTNTGNDVVGVGR^1368^.L	132	1.00E^-11^
	S24	N.^1353^**L**APNTNTGNDVVGVGR^1368^.L	118	6.30E^-10^
	S25	L.^1354^**A**PNTNTGNDVVGVGR^1368^.L	104	4.00E^-09^
	S26	A.^1355^**P**NTNTGNDVVGVGR^1368^.L	108	9.70E^-10^
	S27	P.^1356^**N**TNTGNDVVGVGR^1368^.L	74	9.20E^-06^
	S28	N.^1357^**T**NTGNDVVGVGR^1368^.L	85	3.40E^-06^
	S29	T.^1358^**N**TGNDVVGVGR^1368^.L	80	1.90E^-06^
	S30	N.^1359^**T**GNDVVGVGR^1368^.L	59	3.00E^-04^
	S31	T.^1360^**G**NDVVGVGR^1368^.L	46	2.10E^-03^
	S32	G.^1361^**N**DVVGVGR^1368^.L	52	4.40E^-03^
15	S33	D.^1447^**I**PASVNPKMVR^1457^.L	62	2.80E^-05^
		C-terminal semi-tryptic peptides		
2		R.^137^ALYDLDFSKLNPQTPTRDQTGQITFNPF**G**^165^.G	35	0.001
4		R.^224^TESGQNTSTTGAMFGLKVKNAEA**D**^247^.T	102	3.70E^-09^
5		R.^386^TAIDRVDHL**D**^395^.P	38	6.40E^-03^
9		R.^224^NDKASSGQSDENHTKFTSATGMDQQGQSGTSAGNPDSLKQDNISKS**G**^246^.D	57	1.10E^-05^
		R.^224^NDKASSGQSDENHTKFTSATGMDQQGQSGTSAGNPDSLKQDNISKSG**D**^247^.S	68	3.50E^-06^
13		R.^1273^QSFGTDHSTQPQPQSLKTTTPVFGTSS**G**^1300^.N	27	6.20E^-03^

Exact site within the peptide is indicated by the bold underlined amino acid. Amino acid positions denote the start and end of the peptides. The peptides listed are the highest scores identified from 4 biological replicates analysed separately using Sciex 5600 and Thermo Scientific Q Exactive™ mass spectrometers. All peptides have expectation values <0.05.

**Figure 2:**
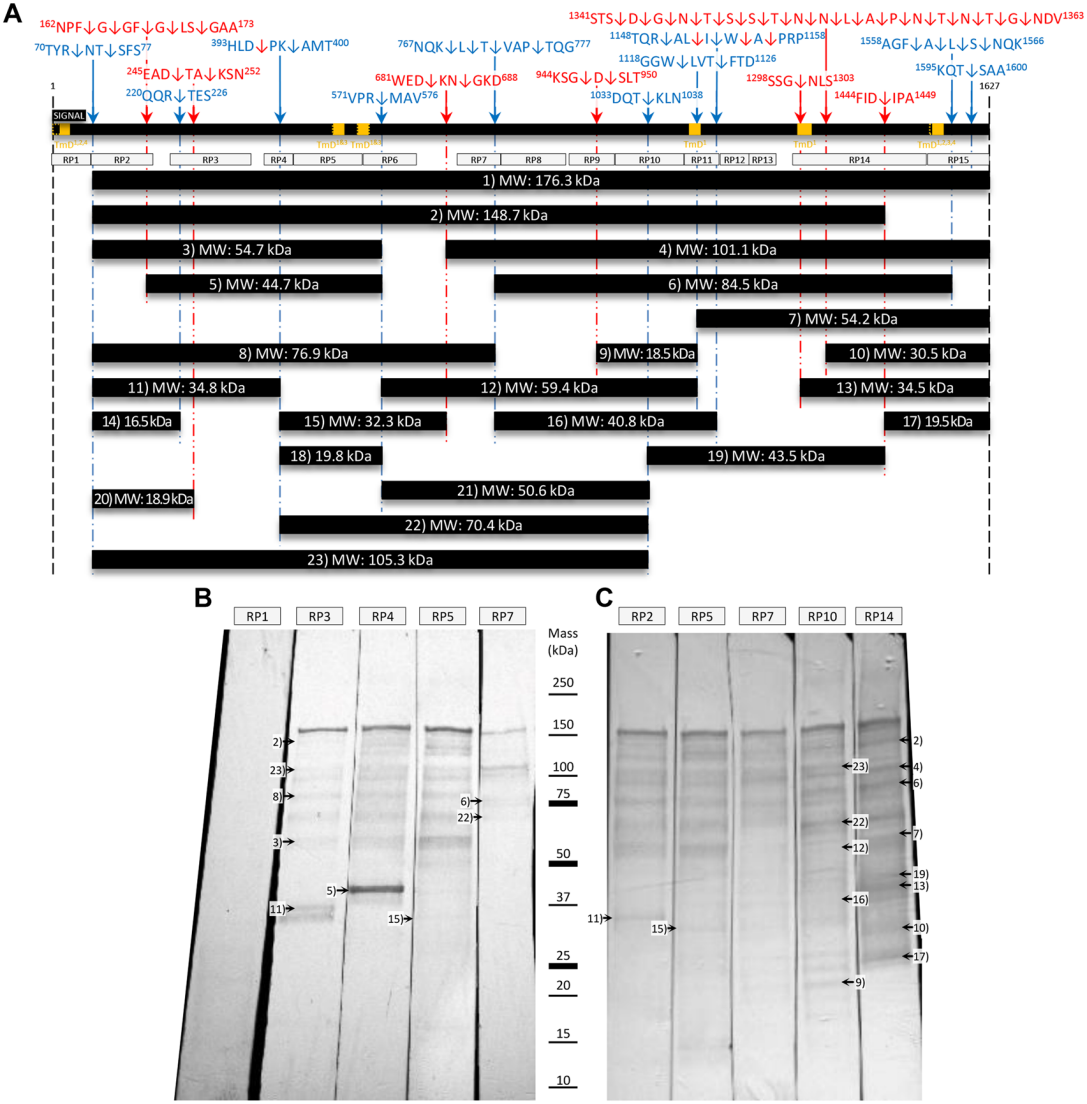
Immunoblots of cell lysates of *M*. *pneumoniae* probed with sera raised against regions within P1. Sera raised against 15 different regions (‘RP’ boxes) of P1 were a gift from R. Dumke^55^. (**Top panel**) Simplified cleavage map depicting the P1 adhesin, cleavage sites, and the 15 regions of P1 that have been previously cloned and expressed as recombinant fragments in *E*. *coli*^55^. The mass of full length and smaller proteoforms of P1 as predicted by ProtParam^65^. (**Bottom panel**) Immunoblots depicting *M*. *pneumoniae* cell lysate probed with the panel of anti-recombinant P1 sera. All the immunoblot lanes are part of the same blot. The membrane was blocked and then sliced to separate lanes before incubating with the described P1 sera. (**Bottom right**) Immunoblots with the intensity adjusted to highlight low abundant bands. Proteins migrating with masses similar to P1 proteoforms identified by LC-MS/MS have been marked on the immunoblot.

Immunoblots of *M*. *pneumoniae* cell lysates probed individually with sera raised against the 15 recombinant regions spanning P1^55^ showed complex banding profiles (Fig. 2). RP1 antiserum that targeted the signal sequence (first 59 amino acids) failed to identify P1 proteoforms suggesting that the signal peptide is destroyed during the early stages of processing of P1 and was also used as a secondary negative control (Fig. 2B). RP3, RP4, RP5, and RP7 span the first half of P1 and the immunoblots detected the full length protein and proteoforms consistent with those representing P1 fragments 1, 2, 5, 7, 10, 13, 21, and 22 (Fig. 2B). P1 fragment 5 was identified in great abundance in RP4 sera, but no band was detected in RP3 or RP5. This could possibly be due to changes to exposed epitopes created from cleavage^76–78^, though further investigation is required. RP2, RP5, RP7 (higher antibody concentration), RP10, and RP14 sera revealed the full length adhesin and P1 fragments 1, 3, 4, 6, 8, 9, 11, 12, 13, 14, 15, 18, 21, and 22 (Fig. 2C). Fragments of P1 that were not identified with confidence were 16, 17, 19, and 20. Data presented in Fig. 2B,C suggest that processing of P1 is complex.

### Functional analysis of the C-terminal tail of P1

**Figure 3:**
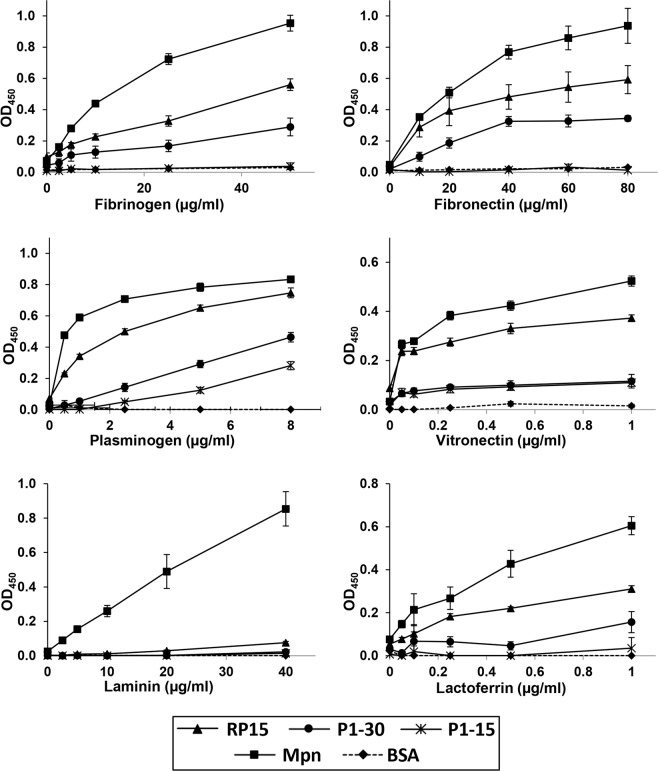
Concentration-dependent binding of the C-terminus of P1 to different human proteins. Microtitre plate binding assays were used to measure the binding abilities of RP15, P1-30, and P1-15 to human plasminogen and to different components of the human extracellular matrix. Bovine serum albumin (BSA) and whole cell lysate proteins of *M*. *pneumoniae* (Mpn) were used as a negative and positive control, respectively. Results are shown from a single experiment with a mean and standard deviation of eight replicates. The experiment was independently repeated twice.

Dimethyl labelling data indicated that the carboxy-terminal 30 residues of P1 is released by a cleavage event at serine^1598^ (cleavage site 17 in Table 1, sequence: ^1595^KQT↓SAA^1600^). The C-terminal peptide has a composition comprising five alanine, five lysine, and thirteen proline residues. This C-terminal region also shares sequence identity (53.1%) with the carboxy-terminal 31 residues of Mpn142. Furthermore, the final 15 residues of P1 shares 73.3% sequence identity with the last 14 residues of Mpn142 (11 identical positions). The C-terminal 30 amino acids (named P1-30: ^1597^TSAAKPGAPRPPVPPKPGAPKPPVQPPKKPA^1627^), and the C-terminal 15 amino acids (named P1-15 ^1613^PGAPKPPVQPPKKPA^1627^) were synthesised chemically (Table 2; Chempeptide Limited, China) and an N-terminal biotin tag was added to the P1-15 peptide. Microtitre binding assays revealed that P1-15, P1-30, and the recombinant protein, RP15^55^, bind a range of host molecules in a dose dependent manner (Fig. 3). *M*. *pneumoniae* cells and RP15 bound lactoferrin, vitronectin, plasminogen, fibronectin, and fibrinogen. Only *M*. *pneumoniae* cells bound laminin. P1-30 bound fibronectin, fibrinogen and plasminogen in a dose dependent manner but failed to bind laminin. P1-15 only bound plasminogen in a dose dependent manner but also bound to vitronectin but failed to bind laminin, lactoferrin, fibronectin, and fibrinogen (Fig. 3). Compared with P1-30 and P1-15, the C-terminal 106 amino acids of P1 represented by RP15 consistently showed the most consistent and most diverse binding capabilities for the panel of host proteins tested here suggesting that multiple binding domains increase the binding capabilities of P1 proteoforms. Consistent with this hypothesis, RP15 spans two putative glycosaminoglycan binding motifs (underlined motifs in Table 2) that are absent in P1-30 and P1-15.

**Table 2:** The C-terminal fragments used in this study.

Name	Sequence	Source
P1-15	^1613^PGAPKPPVQPPKKPA^1627^	This study
P1-30	^1597^TSAAKPGAPRPPVPPKPGAPKPPVQPPKKPA^1627^	This study
RP15	^1521^DYVLPLAITVPIVVIVLSVTLGLAIGIPMHKNKQALKAGF ALSNQKVDVLTKAVGSVFKEIINRTGISQAPKRLKQTSAAKPGAPRPPVPPKPGAPKPPVQPPKKPA^1627^	^55^

**Figure 4:**
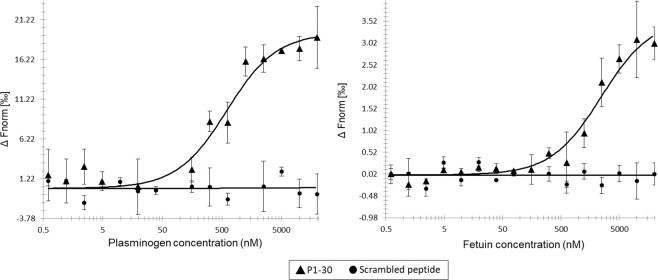
Plasminogen and fetuin binding by P1-30 using microscale thermophoresis. **Left:** Thermophoretic output representing P1-30 (triangles) binding to plasminogen with a K_D_ of 554 nM. A scrambled version of P1-30 (circles) could not be assigned a K_D_ value. **Right:** Thermophoretic output representing P1-30 binding to fetuin with a K_D_ of 2 μM. The scrambled peptide could not be assigned a K_D_ value.

To investigate whether binding was due to the specific amino acid sequence or to amino acid composition, microscale thermophoresis was performed on P1-30 and a scrambled version of P1-30 (PKPPRAAPPKAPTPVPPGPASPVKKPKQAPG). P1-30 had a medium binding affinity for plasminogen (K_D_ = 554 ± 2.1 nM) and a medium/low binding affinity for fetuin (K_D_ = 2.4 ± 0.7 μM). No binding affinity could be detected for the scrambled peptide (Fig. 4).

**Figure 5:**
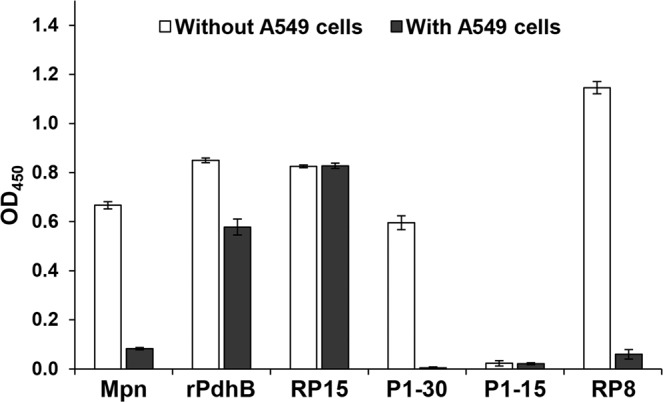
Binding of the C-terminus of P1 to immobilized A549 cells. Proteins, peptides, or A549 cells were immobilised in wells of a 96-well plate and binding was measured in a microtitre plate binding assay. ‘Without A549’ cells: protein and peptides were immobilised and detected by corresponding antisera (peptides with anti-RP15). ‘With A549 cells’: A549 cells were immobilised first before incubating with proteins and peptides. Whole antigen of *M*. *pneumoniae* (Mpn), recombinant PdhB, and RP8 served as positive and negative controls, respectively^17^. Bars represent mean and standard deviation of eight replicates from a single experiment. The experiment was independently repeated twice.

Microtitre binding assays were also employed to determine the binding capabilities of regions spanning the C-terminus of P1 to A549 human epithelial cells (Fig. 5). Recombinant pyruvate dehydrogenase subunit B of *M*. *pneumoniae* (rPdhB; positive control^16^) and RP15 bound immobilised A549 cells, but not P1-30. We were not able to determine if P1-15 bound using this assay because we lacked reagents that could detect this peptide.

To overcome this experimental limitation and to attempt to identify potential binding partners for P1-15, we designed an affinity bait-prey experiment. The biotinylated P1-15 was coupled to avidin agarose and, in parallel with uncoupled avidin-agarose (negative control), were exposed to a native A549 cell lysate as described in Methods, washed and eluants were characterised by SDS-PAGE and LC-MS/MS (Fig. S1). Three protein bands identified in eluents from avidin-agarose coupled with biotinylated P1-15 that were absent in the control were analysed by LC-MS/MS (Fig. S1). LC-MS/MS analysis of slice 1 and 2 identified tryptic peptides that mapped to the intermediate filament cytoskeletal proteins cytokeratin 7 (Mascot score = 1157), cytokeratin 8 (Mascot score = 2737 & 1486), cytokeratin 18 (Mascot score = 2592), and vimentin (Mascot score = 617) (Fig. S1). Tryptic peptides to these filament proteins were not identified in the control experiment. Tryptic peptides identified in slice 3 identified glyceraldehyde-3-phosphate dehydrogenase, however, this protein was also identified in the eluents from the control and was not considered further as a potential binding partner with P1-15.

## Discussion

*M*. *pneumoniae* binds diverse host cell proteins including plasminogen, fibronectin, vitronectin, fibrinogen, lactoferrin, glycosaminoglycans, and sialoglyconjugates^9–17^.

The P1 adhesin and proteins it associates with at the tip of the attachment organelle are central to binding interactions that enable *M*. *pneumoniae* to target host cell receptors and is likely to contain binding domains for some or all of these host molecules. Here we show that Mpn141 is processed extensively generating 23 proteoforms and that many proteoforms are retained on affinity matrices loaded with different host molecules and mimics of regions of host proteins including fetuin, fibronectin, actin, heparin, and plasminogen. Microtitre plate binding assays and microscale thermophoresis assays confirmed several of these preliminary findings and showed that the C-terminal region of P1 binds vitronectin, fibrinogen and fibronectin. Apart from removal of a 59 amino acid N-terminal leader peptide, only a ~40 kDa carboxyl terminal truncated fragment of P1 (potentially representing fragment 18 from this study), that forms a complex with full length P1 protein, and other accessory proteins has been reported previously^33^ but earlier immunoblotting studies with anti-P1 monospecific antisera identified numerous smaller proteoforms of P1 that were not characterised^2^. Dimethyl labelling experiments enabled us to map the precise location of cleavage events in P1 (Table 1). P1 proteoforms are likely generated by proteases on the cell surface of *M*. *pneumoniae* or associated with the protein translocation machinery but their identities have not been confirmed. Biotinylation studies identified 13 proteoforms of P1 that were accessible on the surface of *M*. *pneumoniae* and our surface labelling and trypsin shaving experiments indicate that the proteoforms remain attached to the extracellular side of *M*. *pneumoniae* cell membranes. Our data is consistent with electron micrographs of *M*. *pneumoniae* immunostained with ferretin-labelled anti-P1 antibodies that depict gold particles at: i) the tip of the attachment organelle; ii) along the shaft of this structure; iii) at sites along the cell body; and iv) at sites distant from the *M*. *pneumoniae* membrane^2^. It is not known if some proteoforms are excreted into the extracellular milieu but it is conceivable that processing of P1 occurs after translocation and the fragments may remain anchored to the surface via the predicted C-terminal transmembrane domain similarly seen in P40 and P90 of *M. pneumoniae*^27^. Consistent with this view, we were unable to find tryptic peptides that mapped to the putative leader peptide residing in the N-terminus of P1 or in the bioinformatically predicted transmembrane domains, or the well characterised C-terminal transmembrane domain. However, we did find tryptic peptides in the bioinformatically predicted  transmembrane domain located around residue 1294.

Regions in P1 have been extensively characterised in an earlier study^55^. Highly immunogenic regions and adherence mediating regions were found distributed throughout P1 particularly in the carboxy-terminal half of the molecule^55^. Sera from patients infected with *M*. *pneumoniae* bound to regions in P1 that were not responsible for adherence^55^. It is conceivable that P1-derived proteoforms divert the binding of host antibodies away from regions in P1 required for adherence. We hypothesise that post-translational processing events release a proportion of P1-derived proteoforms into the extracellular milieu, a process that may represent an immune decoy mechanism that seeks to bind and direct host antibodies away from *M*. *pneumoniae*. A similar scenario has been hypothesized for Protein M of *Mycoplasma genitalium*; a close relative of *M*. *pneumoniae*^79^.

Our affinity studies suggest that the different proteoforms retain the ability to bind to different host proteins, glycosaminoglycans and sialoglyconjugates. RP15 was observed to bind immobilised A549 cells in microtitre plate assays (Fig. 4). This was surprising as no adherence regions have been previously identified within RP15. Anti-RP15 antibodies were reported to be unable to inhibit *M*. *pneumoniae* adherence to primary human bronchial epithelial (HBEC) cells, human fetal lung fibroblasts (MRC-5), and human cervical carcinoma cells (HeLa)^55^ suggesting that RP15 may bind to specific receptors only present on the A549 cell surface. We were unable to determine binding activity to A549 cells for P1-30 or P1-15 (Fig. 4) because anti-RP-15 antibodies did not detect these peptides. To investigate the binding capabilities of the C-terminal peptide P1-15, it was bound to avidin agarose and incubated with A549 cell lysates. This strategy selectively recovered cytoskeletal proteins, vimentin, cytokeratin 7, cytokeratin 8, and cytokeratin 18 (Fig. S1) from P1-15-avidin agarose but not from avidin agarose control experiments. Although preliminary, these observations are worthy of further study. Cytokeratin 7 is found in epithelia of lungs and other tissues^80^, and has been shown to be involved in stabilising cytokeratin 18^81^. Both cytokeratin 8 and 18 are major structural proteins of epithelial cells^82^ and are found in the intermediate filaments of A549 cells^83^. Cytokeratin 8 has been identified to reside on the cellular surface of carcinogenic keratinocyte cells (HaCat)^84^, carcinogenic mammary cells^85^, and carcinogenic hepatocytes^86^ suggesting they may be surface accessible on many cells. Cytokeratin 8 and 18 are co-expressed and frequently found associated together^87,88^. Vimentin forms filaments and is primarily expressed when epithelial cells transition into mesenchymal cells and function to induce changes in cell shape, motility and adhesin during this transition^89,90^. Vimentin has also been observed to be secreted to the extracellular matrix and on the surface of activated macrophages^91^. Cytokeratin 8, 18, and vimentin are suggested to be targeted by different pathogens after successfully invading host cells^84,92–95^ or after inducing cytoskeletal rearrangement^96–100^. Pathogenic bacteria are known to interact with these cytoskeletal proteins during infection^95,101,102^. Although mycoplasma have long been considered to be cell surface-associated parasitic bacteria, this dogma has been challenged with numerous reports citing phylogenetically-divergent mycoplasmas residing within eukaryote cells and possessing the molecular machinery for selective uptake into, survival within, and release from phagosomes^103–109^.

We recently showed that Mpn142, a member of the same operon that houses the P1 gene (*mpn141*), and the surface accessible moonlighting adhesin, elongation factor Tu (Ef-Tu), are cleaved extensively^12,28^. Post-translational processing of adhesins has been well characterised in *M*. *hyopneumoniae* where cleavage fragments have been shown to adhere to porcine cilia, porcine kidney epithelial cells, and a range of host molecules such as the glycosaminoglycan mimic heparin^59–62,64,72,74,110–115^, plasminogen^60,112–114^, actin^116^, and fibronectin^59,72,112–114^. Processing of adhesin molecules is not confined to *M*. *hyopneumoniae* but has been described in *Mycoplasma gallisepticum*^117^, *Mycoplasma fermentans*^118–120^, *M*. *genitalium*^121^, and *Spiroplasma citri*^122^. Here we show that major adhesion molecules in *M*. *pneumoniae*, a phylogenetically distinct human pathogen, are processed^12,28^. All these studies suggest that the processing of surface accessible proteins is widespread in Mollicutes. It is notable that all the P1 fragments that were recovered during heparin affinity chromatography contained putative glycosaminoglycan binding motifs except an N-terminal and a central fragment (Fig. 1, fragments 14 and 16). These motifs consist of clustered, positively charged amino acids that have been shown to have a role in binding to glycosaminoglycans^69,72^, actin^123^, and plasminogen^123^. Heparin mimics the glycosaminoglycans found in the extracellular matrix and on the surface of host cells^124^. *M*. *hyopneumoniae*, and *M*. *gallisepticum* have been shown to bind heparin to aid in host adherence^110,125^. Pathogens such as *Staphylococcus* and *Neisseria* spp., *Helicobacter pylori*, and *Streptococcus pyogenes* are able to recruit heparin to the bacterial cell surface and employ bound heparin to bind other host molecules^126^. Finally, heparin has also been implicated in biofilm formation by increasing cell-cell interactions in the Gram-positive pathogens, *S*. *aureus*^127^ and *Lactobacillus rhamnosus*^128^. *M*. *pneumoniae* forms large, complex biofilms on abiotic surfaces^34^. Heparin affinity chromatography of *M*. *pneumoniae* has been performed previously^129^ identifying only nine proteins, none of which was P1. Recently, we showed that Ef-Tu in *M*. *pneumoniae* displays a strong affinity to heparin^12^. Collectively, our studies suggest that the ability to bind heparin is a universal strategy in microbial pathogenesis.

In several instances, we observed multiple cleavage sites within P1 that clustered within a defined region of P1. For example, 18 cleavage sites clustered between amino acids 1343–1361 in the C-terminus of P1 (Table 1). Sequential cleavage patterns similar to this was also reported in Mpn142^28^ and in Mhp493, a paralog of the major adhesin P97 (Mhp183) in *M*. *hyopneumoniae*^74^. Surfaceome studies of *M*. *pneumoniae* (data not shown) revealed the presence of surface accessible aminopeptidases that may target a neo-N-terminal cleavage event and sequentially clip amino acids subsequent to the initial cleavage event. The function of these clipping events remains unknown but could be a mechanism to alter function and localisation of cleavage fragments, or represent a mechanism to recycle amino acids^74^. Cleavage site 14 in P1 (Fig. 1) occurs within a large predicted disordered region (amino acid range 1187–1382). The inherent flexibility of disordered regions make them accessible to protease activity^130^. Many major cleavage events identified in *M*. *hyopneumoniae* adhesin molecules reside with large disordered regions^60–62,74,114,115^.

The C-terminus of the P1 tail is homologous to the C-terminus of Mpn142 and the C-terminal 15 amino acids of P1 (^1613^PGAPKPPVQPPKKPA^1627^) has 73.3% sequence identity with the same region in Mpn142. Almost half of this sequence consists of proline residues while lysine is also heavily represented in this region. Proline-rich regions in proteins have been implicated in protein:protein interactions^131–133^ and it has been suggested that proline residues could anchor the C-terminus of P1 in the cell membrane^49^. Lysine-rich regions are associated with binding plasminogen^60,64,123,134,135^, heparin^59,61,69,72,115,136^, actin^116,123^, and DNA^75,137^. While P1-15 and P1-30 bound plasminogen in a dose-responsive manner, it was notable that RP-15 bound it more strongly. RP-15 also bound fibronectin and fibrinogen more strongly than P1-30 (Fig. 3). These data suggest that extra binding sites for these host molecules are located upstream of the C-terminal 30 amino acids of P1. Previous work suggests that sialic acid is the dominant host receptor for the P1 adhesin^18–21^. Consistent with these earlier studies the P1 tail has a strong affinity to the sialic acid rich protein, fetuin. Our data indicates that the mature P1 proteoform and a further nine smaller proteoforms of P1 bind fetuin. The ability to bind fetuin has been linked with biofilm formation in *M*. *pneumoniae*^34^.

## Conclusion

In summary, this study reports that the P1 adhesin is subject to extensive post-translational processing forming twenty-two proteoforms from seventeen cleavage sites. Each of the proteoforms retain the ability to bind to host molecules or their structural mimics and are surface accessible. Processing has been described in *M*. *hyopneumoniae*, *M*. *gallisepticum*, and *S*. *citri* and is likely to be a widespread mechanism to generate surface protein diversity and promote protein:protein interactions. Specifically we show that the C-terminus of P1 plays a role in adhering to a range of host molecules including cytoskeletal proteins. This study expands on our knowledge of the role that the P1 adhesin plays in interactions between *M*. *pneumoniae* and host cells.

## Supplementary information

Supplementary Information. 

## Data Availability

Data for this study is available on request from the corresponding author.
