# Supplementary material for: Cell surface processing of the P1 adhesin of Mycoplasma pneumoniae identifies novel domains that bind host molecules
Source: Sci Rep. 2020 Apr 14;10:6384. doi: 10.1038/s41598-020-63136-y (PMC7156367; doi:10.1038/s41598-020-63136-y)
Supplement: Supplementary file 1 — Supplementary Information. [file 41598_2020_63136_MOESM1_ESM.pdf]

Cell surface processing of the P1 adhesin of *Mycoplasma pneumoniae* identifies novel domains that bind host molecules

Michael Widjaja[1], Iain James Berry[1], Veronica Maria Jarocki[1], Matthew Paul Padula[3], Roger Dumke[2¥], Steven Philip Djordjevic[1,3,¥*]

[1] The ithree institute, University of Technology Sydney, PO Box 123, Broadway, NSW, 2007, Australia.

[2] Technische Universität Dresden, Medizinische Fakultät Carl Gustav Carus, Institut für Medizinische Mikrobiologie und Hygiene, Fetscherstrasse 74, 01307 Dresden, Germany.

[3] Proteomics Core Facility and School of Life Sciences, University of Technology Sydney, PO Box 123, Broadway, NSW, 2007, Australia.

¥ These contributors share senior authorship

*Corresponding Author:

Prof. Steven P. Djordjevic

The ithree institute, University of Technology Sydney. PO Box 123, Broadway, NSW, 2007, Australia

Phone: +612 9514 4127

Fax: +612 9514 4143

Email: Steven.Djordjevic@uts.edu.au

Supplementary Figure

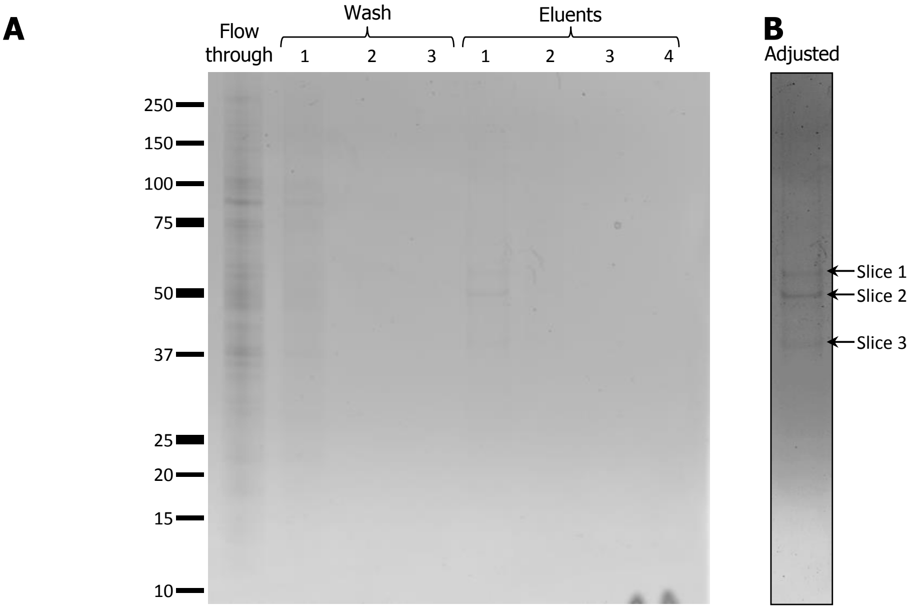

C

Slice 1

```
P05787|Keratin, type II cytoskeletal 8
  1 MSIRVTQKSY KVSTSGPRAF SSRSYTSGPG SRISSSSFSR VGSSNFRGGL
 51 GGGYGGASGM GGITAVTVNQ SLLSPLVLEV DPNIQAVRTQ EKEQIKTLNN
101 KFASFIDKVR FLEQQNKMLE TKWSLLQQQK TARSNMDNMF ESYINNLRRQ
151 LETLGQEKLK LEAELGNMQG LVEDFKNKYE DEINKRTEME NEFVLIKKDV
201 DEAYMNKVEL ESRLEGLTDE INFLRQLYEE EIRELQSQIS DTSVVLSMDN
251 SRSLDMDSII AEVKAQYEDI ANRSRAEAES MYQIKYEELQ SLAGKHGDDL
301 RRTKTEISEM NRNISRLQAE IEGLKGQRAS LEAAIADAEQ RGELAIKDAN
351 AKLSELEAAL QRAKQDMARQ LREYQELMNV KLALDIEIAT YRKLLEGEES
401 RLESGMQNMS IHTKTTSGYA GGLSSAYGGL TSPGLSYSLG SSFGSGAGSS
451 SFSRTSSSRA VVVKKIETRD GKLVSESSDV LPK
```

```
P08729|Keratin, type II cytoskeletal 7
  1 MSIHFSSPVF TSRSAAFSGR GAQVRLSSAR PGGLGSSSLY GLGASRPRVA
 51 VRSAYGGPVG AGIREVTINQ SLLAPLRLDA DPSLQRVRQE ESEQIKTLNN
101 KFASFIDKVR FLEQQNKLLE TKWTLLQEQK SAKSSRLPDI FEAQIAGLRG
151 QLEALQVDGG RLEAELRSMQ DVVEDFKNKY EDEINHRTAA ENEFVVLKKD
201 VDAAYMSKVE LEAKVDALND EINFLRTLNE TELTELQSQI SDTSVVLSMD
251 NSRSLDLDGI IAEVKAQYEE MAKCSRAEAE AWYQTKFETL QAQAGKHGDD
301 LRNTRNEISE MNRAIQRLQA EIDNIKNQRA KLEAAIAEAE ERGELALKDA
351 RAKQEELEAA LQRGKQDMAR QLREYQELMS VKLALDIEIA TYRKLLEGEE
401 SRLAGDGVGA VNISVMNSTG GSSSGGGIGL TLGGTMGSNA LSFSSSAGPG
451 LLKAYSIRTA SASRRSARD
```

```
P08670|Vimentin
  1 MSTRSVSSSS YRRMFGGPGT ASRPSSSRSY VTTSTRTYSL GSALRPSTSR
 51 SLYASSPGGV YATRSSAVRL RSSVPGVRLL QDSVDFSLAD AINTEFKNTR
101 TNEKVELQEL NDRFANYIDK VRFLEQQNKI LLAELEQLKG QGKSRLGDLY
151 EEEMRELRRQ VDQLTNDKAR VEVERDNLAE DIMRLREKLQ EEMLQREEAE
201 NTLQSFRQDV DNASLARLDL ERKVESLQEE IAFLKKLHEE EIQELQAQIQ
251 EQHVQIDVDV SKPDLTAALR DVRQQYESVA AKNLQEAEEW YKSKFADLSE
301 AANRNNDALR QAKQESTEYR RQVQSLTCEV DALKGTNESL ERQMREMEEN
351 FAVEAANYQD TIGRLQDEIQ NMKEEMARHL REYQDLLNVK MALDIEIATY
401 RKLLEGEESR ISLPLPNFSS LNLRETNLDS LPLVDTHSKR TLLIKTVETR
451 DGQVINETSQ HHDDLE
```

Slice 2

```
P05783|Keratin, type I cytoskeletal 18
  1 MSFTTRSTFS TNYRSLGSVQ APSYGARPVS SAASVYAGAG GSGSRISVSR
 51 STSFRGGMGS GGLATGIAGG LAGMGGIQNE KETMQSLNDR LASYLDRVRS
101 LETENRRLES KIREHLEKKG PQVRDWSHYF KIIEDLRAQI FANTVDNARI
151 VLQIDNARLA ADDFRVKYET ELAMRQSVEN DIHGLRKVID DTNITRLQLE
201 TEIEALKEEL LFMKKNHEEE VKGLQAQIAS SGLTVEVDAP KSQDLAKIMA
251 DIRAQYDELA RKNREELDKY WSQQIEESTT VVTTQSAEVG AAETTLTELR
301 RTVQSLEIDL DSMRNLKASL ENSLREVEAR YALQMEQLNG ILLHLESELA
351 QTRAEGQRQA QEYEALLNIK VKLEAEIATY RRLLEDGEDF NLGDALDSSN
401 SMQTIQKTTT RRIVDGKVVS ETNDTKVLRH
```

```
P05787|Keratin, type II cytoskeletal 8
  1 MSIRVTQKSY KVSTSGPRAF SSRSYTSGPG SRISSSSFSR VGSSNFRGGL
 51 GGGYGGASGM GGITAVTVNQ SLLSPLVLEV DPNIQAVRTQ EKEQIKTLNN
101 KFASFIDKVR FLEQQNKMLE TKWSLLQQQK TARSNMDNMF ESYINNLRRQ
151 LETLGQEKLK LEAELGNMQG LVEDFKNKYE DEINKRTEME NEFVLIKKDV
201 DEAYMNKVEL ESRLEGLTDE INFLRQLYEE EIRELQSQIS DTSVVLSMDN
251 SRSLDMDSII AEVKAQYEDI ANRSRAEAES MYQIKYEELQ SLAGKHGDDL
301 RRTKTEISEM NRNISRLQAE IEGLKGQRAS LEAAIADAEQ RGELAIKDAN
351 AKLSELEAAL QRAKQDMARQ LREYQELMNV KLALDIEIAT YRKLLEGEES
401 RLESGMQNMS IHTKTTSGYA GGLSSAYGGL TSPGLSYSLG SSFGSGAGSS
451 SFSRTSSSRA VVVKKIETRD GKLVSESSDV LPK
```

Figure S1: LC-MS/Ms analysis of eluants from affinity experiments using avidin-agarose coupled with biotinylated P1-15. SDS-PAGE of the chromatography flow through, washes, and eluents. The full length Coomassie stained gel can

be seen in Figure S2. B) Same eluent 1 as panel (A) but transformed to highlight the three protein bands analysed by LC-MS/MS. C) Tryptic peptides identified by LC-MS/MS of slices 1 and 2 are underlined.

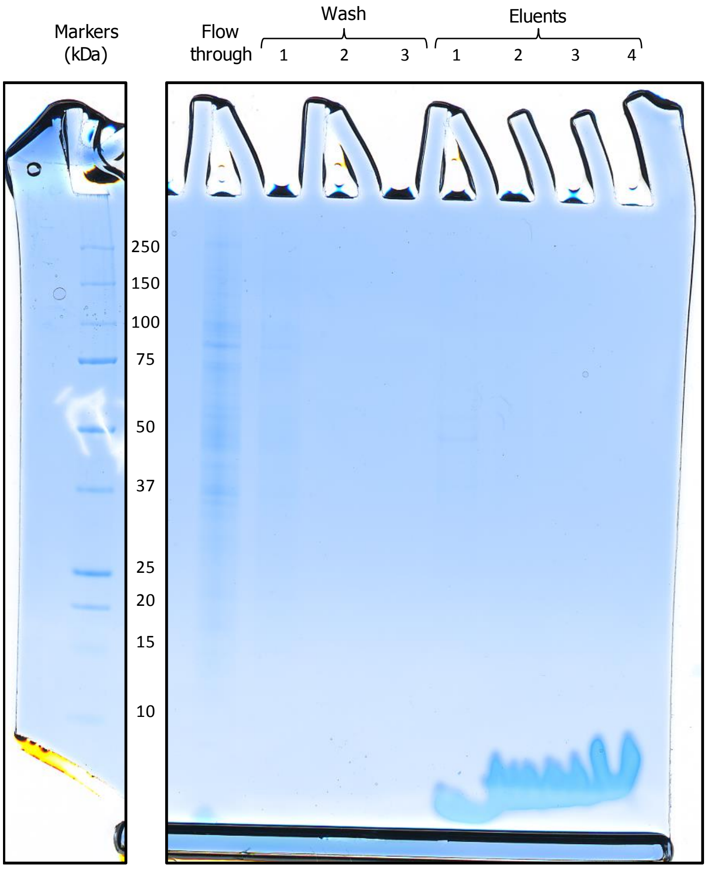

Figure S2: Full length SDS-PAGE of the affinity chromatography experiments using avidin-agarose coupled with biotinylated P1-15. The image here has not been edited or transformed. The Markers are from the same gel along with the flow through, washes, and eluents.
